# Age, Motor Function, and Cognitive Function Influence Preferences for Telerehabilitation Mediated by a Social Robot Augmented With Telepresence

**DOI:** 10.1109/TNSRE.2025.3592020

**Published:** 2025

**Authors:** Michael J. Sobrepera, Anh T. Nguyen, Ajay Anand, Laura A. Prosser, Sally H. Evans, Michelle J. Johnson

**Affiliations:** Department of Mechanical Engineering and Applied Mechanics, University of Pennsylvania, Philadelphia, PA 19104 USA. He is now with Boston Consulting Group, Boston, MA 02210 USA.; Department of Bioengineering, University of Pennsylvania, Philadelphia, PA 19104 USA.; Rehabilitation Robotics Laboratory, Philadelphia, PA 19146 USA. He is now with the Department of Mechanical Engineering and the Robotics Center, The University of Utah, Salt Lake City, UT 84112 USA.; Department of Pediatrics, University of Pennsylvania, Philadelphia, PA 19104 USA, and also with the Children’s Hospital of Philadelphia, Philadelphia, PA 19104 USA.; Department of Pediatrics, University of Pennsylvania, Philadelphia, PA 19104 USA, and also with the Children’s Hospital of Philadelphia, Philadelphia, PA 19104 USA.; Departments of Physical Medicine and Rehabilitation, Bioengineering, and Mechanical Engineering and Applied Mechanics, University of Pennsylvania, Philadelphia, PA 19104 USA, and also with the Rehabilitation Robotics Laboratory, The GRASP Laboratory, Philadelphia, PA 19104 USA.

**Keywords:** Children, cognition, motor control, older adults, social robots, telerehabilitation, upper limb

## Abstract

Social robot augmented telepresence (SRAT) is a potential approach to provide rehabilitative care to remote patients, while overcoming barriers to physical clinician-patient interaction. This study evaluated the preference of the subjects, stratified by age, motor impairment level and cognitive impairment level, for three modes of rehabilitation care delivery: face-to-face (FTF), classical telepresence (CT), and via social robot-augmented classical telepresence (SRAT). Forty-two participants completed the experiment that included assessments of upper-limb motor function and cognitive function followed by simulated rehabilitation interaction sessions, where the FTF interaction was the first, followed by CT and SRAT interactions in randomized order. Participants completed surveys on their impression and experience receiving simulated care in each mode. Survey responses were analyzed using descriptive statistics and regression methods. Although in-person interaction (FTF) was the preferred option, 71% of subjects enjoyed and preferred SRAT over CT and this preference was mediated by age and severity of motor and cognitive impairment. Our analysis suggests that young children will rank SRAT above CT except for when they have severe cognitive impairment, adults will prefer SRAT less as their upper-limb impairment becomes more severe, and adults over 70 years old will prefer SRAT less if they have moderate to no upper-limb motor impairment and no cognitive impairment.

## I. Introduction

Individuals with disabilities have historically faced barriers to accessing rehabilitative care, a challenge further compounded by the shortage and unequal distribution of rehabilitation professionals. [1]. To combat this, new methods of care delivery have been proposed to improve affordability, availability, and equitable access without compromising quality and effectiveness [2]. An increasingly used approach is telerehabilitation (TR), a subset of telehealth that provides rehabilitation services remotely through telecommunication technologies. TR offers a promising approach to expand geographic access and provide consistent rehabilitation services to patients who require frequent care. [3].

Telepresence systems that facilitate telehealth are available in many healthcare settings. They consist of devices with a screen, camera, and internet connectivity. Some commercialized systems from Double Robotics and VGo Communications are further equipped with a mobile robotic base that allows remote control and navigation. There have been numerous successful implementations of telepresence in rehabilitation [4], [5]. For instance, a tele-physical therapy program for post-knee arthroplasty was tested, with 143 participants receiving virtual care and 144 receiving in-person care [4]. Although telepresence offers a wide range of benefits, there are limitations such as reduced presence, limited spatial reasoning, and a lack of physical embodiment that can hinder engagement and therapeutic efficacy [6], [7].

Augmenting TR with socially assistive robots (SARs) may address limitations inherent in telepresence systems and enhance the quality of remote interactions. SARs [8] combine assistive robots, which support users with impairments, with social robots, which are designed to interact and communicate with humans. Through social interactions, SARs can enhance communication and, potentially improve rehabilitation outcomes and motor assessments. For example, Mann et al. [9] compared responses to a physical robot with responses to a remote tablet during an interaction and found that subjects engaged more, responded more positively to the physical robot, followed the robot’s instructions better and found the robot more likable and trustworthy. Bainbridge et al. [10] showed that having a physical presence for interactions is critical for the trust and motivation of the user, especially for tasks that cause discomfort. Additionally, Kiesler et al. [11] also showed that subjects who interact physically with a robot are more engaged and comply better with instructions compared to interacting with a virtual robot.

From our previous work, we know that therapists are optimistic about the addition of social robots in telepresence scenarios with an increase in patient motivation and compliance compared to traditional TR [12]. While these results support that social rehabilitation robots can significantly enhance the patient TR experience, collecting patient feedback after SARs use is crucial for assessing acceptability, feasibility and improving future care quality and efficacy.

Cognitive impairment and motor impairment are key factors that affect acceptance, feasibility, and preference for robot-assisted rehabilitation. Higher cognitive impairment significantly hinders recovery due to communication difficulties and challenges in following instructions, reducing preference and acceptance of robot-aided therapy [13], [14]. Patients with speech impairments often have difficulty interacting with SARs during therapy, as they may struggle to understand the robot’s intentions and communicate effectively [15]. Based on the literature, severe motor impairments strongly influences the rate and extent of recovery and the user’s preference for a system [16]. Age also significantly affects acceptance, feasibility, and preference for robot-assisted rehabilitation. Older adults, in particular, may be slower to adopt new technologies such as robots due to concerns that assistive devices mean dependency and fragility [17]. Older adults with cognitive impairment are a key target group for SARs due to their need for extensive care. Despite barriers such as cost, operational difficulties, distrust, fears of replacing human interaction, and reluctance to accept help (especially in dementia cases), studies show promising results when robots are sophisticated enough to meet the specific needs of these patients [18], [19], [20], [21]. However, it remains unclear how the combination of age, motor function, and cognitive function influences the perceived effectiveness and the preference for social robot augmented telerehabilitation over traditional methods.

**Fig. 1.**
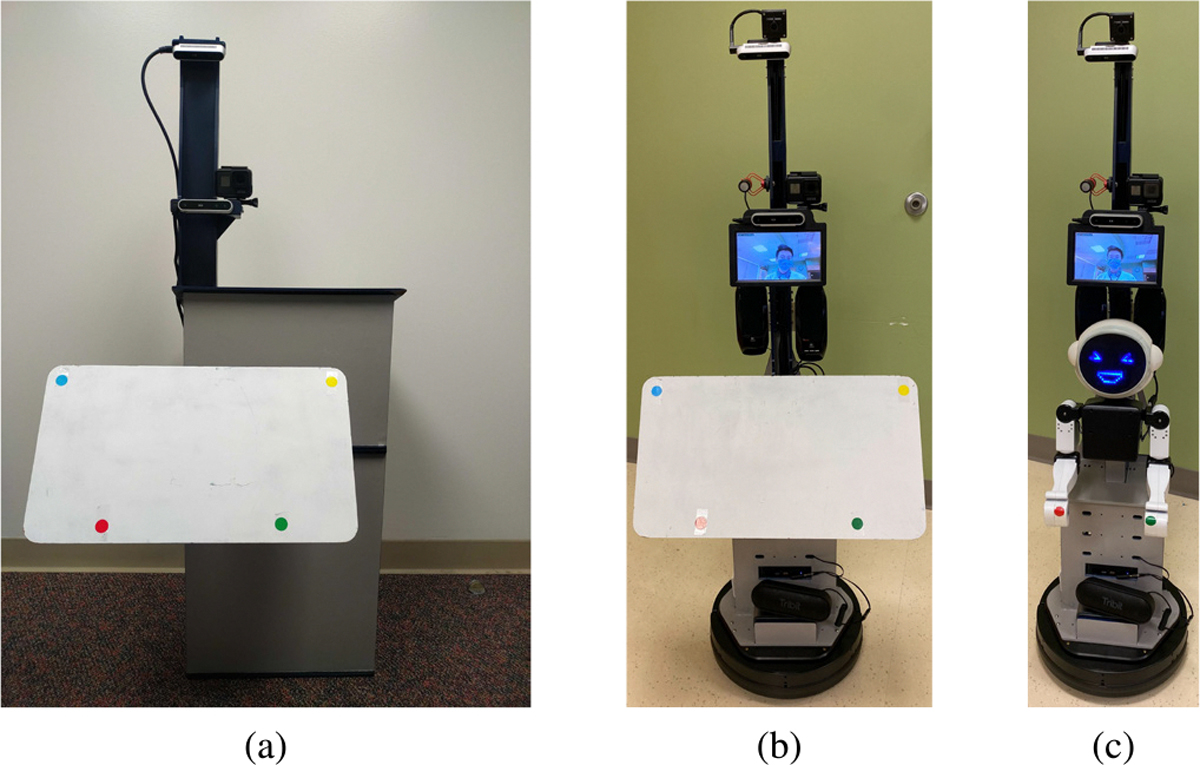
Different configurations for the Flo telepresence platform used throughout the study. (a) Face to face (FTF). (b) Classical telepresence (CT). (c) Social robot augmented telepresence (SRAT).

Telepresence and social robotics, when applied independently in healthcare, each have limitations. To address these limitations, we propose Social Robot Augmented Telepresence (SRAT), which integrates traditional telepresence with a social robot to engage patients in interactive games and guided activities, supervised by a remote clinician. To assess the feasibility of this paradigm, we created an SRAT system, namely Flo (fig. 1c). The details of the system design can be found in [22].

This paper examines whether telerehabilitation augmented by a social robot enhances perceived interaction quality compared to in-person and classical telepresence rehabilitation. It also investigates how age, upper-limb motor impairment, and cognitive impairment influence preferences for telerehabilitation delivered via classical telepresence (CT) or social robot-augmented telepresence (SRAT). The primary goals of this study are: 1) to demonstrate the feasibility of interactions via SRAT with a wide diversity of subjects, as determined by their ability to complete activities, their perception of safety, and their ability to understand instructions, 2) to determine if interaction quality via SRAT is better than via CT, 3) to determine if people prefer SRAT over CT, 4) to determine if and how age, level of upper arm motor function, and level of cognitive function affect perceptions of interaction quality between SRAT or CT, and 5) to determine if and how age, level of motor function, and level of cognitive function affect preference for SRAT and CT. We hypothesized that:

- **H1**: SRAT would outperform CT as a medium for completing rehabilitation tasks, specifically:
  - **H1tlx**: Participants would experience a lower *task load* when completing activities via SRAT compared to CT.
  - **H1e**: Participants would find interactions via SRAT more *enjoyable* than those via CT.
  - **H1c**: Participants would feel more *competent* when interacting via SRAT than via CT.
  - **H1v**: SRAT interactions would be perceived as having higher *value* compared to CT.
  - **H1p**: Participants would experience less *pressure* during SRAT interactions than during CT interactions.
- **H2**: Across all age groups and levels of upper limb motor function and executive cognitive function, participants would prefer SRAT over CT for rehabilitation activities.

The sub-hypotheses under H1 are labeled based on the specific domain they address. The letter suffixes indicate the respective measures: **H1tlx** (Task Load), **H1e** (Enjoyment), **H1c** (Competence), **H1v** (Value), and **H1p** (Pressure). These measures are detailed in Method Section II-C.5 and analyzed in Results Sections III-B and III-C. The present work adds to the body of work in novel methods of rehabilitation and advocates for the advantages of SRATs by conducting a controlled comparison with more traditional methods of rehabilitation while simultaneously considering the demographic parameters of age, arm motor function and executive function.

## II. Methods

### A. Subjects

Subjects 4 years old and older with or without upper extremity impairment were recruited via relationships with clinicians, public flyers, the Penn and CHOP clinical trial subject databases, subject mailing lists held by the Penn Rehab Robotics Lab, and from a pool of inpatient patients at CHOP in the Division of Rehabilitation Medicine. The participating subjects formed a sample of convenience. Forty-four (44) subjects consented to participate in the study. This study was approved by the University of Pennsylvania Institutional Review Board (IRB No. 830126) on June 29, 2021, and accepted by the Children’s Hospital of Philadelphia (CHOP) Office of Research Compliance (IRB No. 21–018664) on July 9, 2021, which served as the IRB of record for CHOP research activities. Forty-two (42) of 44 subjects completed the trial. One subject dropped out of the study prior to completing either telepresence condition and was therefore excluded. During the telepresence interactions for another subject, a wire broke, preventing the system’s audio from working; so that subject’s trial was also excluded.

Subjects completed a series of clinical assessments followed by three rehabilitation interactions requiring different test platforms. Their reaction to the interactions was recorded throughout by survey instruments. Surveys, questionnaires, and game descriptions are provided in the supplement. The following describes the experiment in detail.

### B. Test Platforms

Three test set-ups were constructed to test the rehabilitation interactions: face-to-face (FTF) shown in (fig. 1a), classical telepresence (CT) shown in (fig. 1b) and social robot augmented telepresence (SRAT) shown in (fig. 1c).

In the FTF interaction condition, the operator used a podium (fig. 1a) painted in the same color scheme as the Flo robot featuring a wooden lectern equipped with two Intel RealSense cameras, a GoPro camera on a vertical arm, a screen, and a target touch board with four colored dots which is used for target touch game (detailed in Section II-C.4). Software on the podium guided the operator’s speech and actions to manage the experiment’s process.

For the SRAT condition, the Flo robotic system was used (fig. 1c) [22]. The robotic system can be broken into a telepresence system with computer vision and an expressive humanoid [22]. The telepresence component features a Kobuki mobile robot base with a custom chassis, equipped with two depth cameras, a fisheye camera, a screen, speakers, and a microphone. The anthropomorphic humanoid Flo, mounted on the base, is easily removable and includes a head, face, torso, and arms for demonstrating human motion and exercise gestures. The robot can synthesize speech, change facial expressions in real-time, and is remotely controlled via a web interface. In SRAT, the operator remains present on the telepresence system’s screen and controlled the robot via the remote web interface. The humanoid’s hands, marked with colored dots, could align with the target touch board’s dots on the podium relative to the ground and cameras.

In the CT condition, the Flo robotic system used only the telepresence platform, without the humanoid mounted (fig. 1b). Instead of the humanoid, a target touch board with four dots was mounted on the platform, aligning with the podium’s dots relative to the ground and cameras. The system is operated through the same web interface as the full Flo system, but includes additional instructions for the operator on what to say. By maintaining the same color scheme, design profiles, and target positioning for activities, the study’s mechanical aspects were well controlled. Additionally, software was used to regulate the operator’s delivery of activities, ensuring consistent interactions.

### C. Experiment

**Fig. 2.**
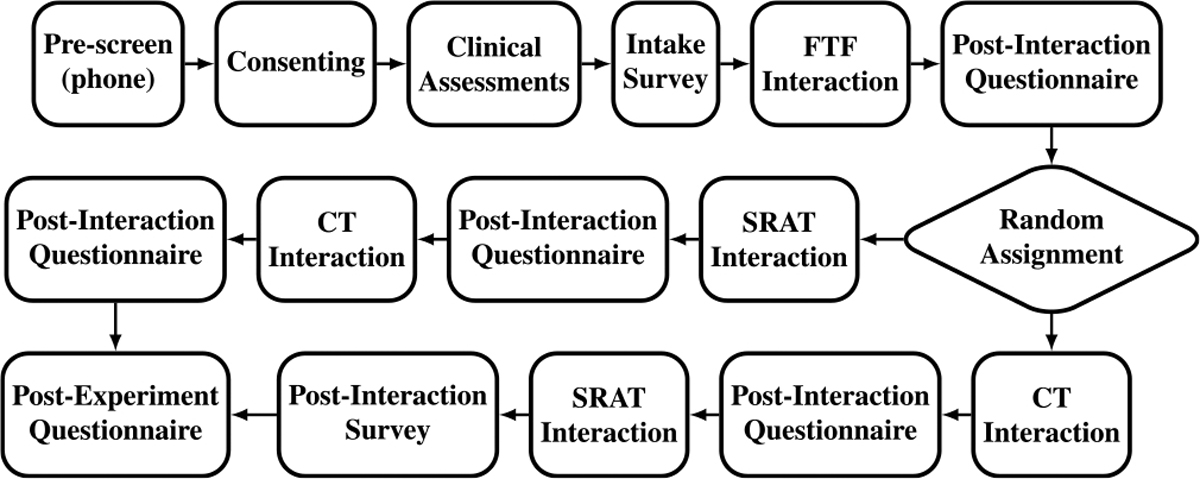
Experiment flow. FTF: Face to face, CT: classical telepresence, SRAT: Social robot augmented telepresence.

Trials took place in one of two environments: 1) a laboratory environment (the Penn Rehab Robotics Lab, the Penn Gait Lab, the CHOP Neuromotor Performance Lab, or the Penn Clinical Simulator) and 2) a clinical environment (the teen activity room in the CHOP Division of Rehabilitation Medicine). Each trial was conducted by a minimum of two researchers: 1) an operator who delivered the rehabilitation activities and operated the robots and 2) an interviewer who administered the subject surveys. For participants in the clinical environment (12 children aged 4–17, mainly inpatients), the trial spanned two days to align with hospital schedules and reduce fatigue. Day 1 included clinical assessments, an intake survey, FTF interactions, and a post-experiment survey. Day 2 covered SRAT and CT interactions, followed by the remaining surveys. In contrast, laboratory environment participants completed the trial in a single two-hour session. The flow of the experiment is shown in fig. 2 and detailed below.

#### 1) Pre-Trial:

Before the trial, all participants underwent a phone pre-screen. Those in the clinical environment were pre-screened by a care team clinician. Participants were then scheduled for an experiment slot and sent a consent form.

#### 2) Clinical Assessments:

After consenting, subjects were assessed using the *Box and Block Test* (BBT) [23], *Color Trails Test 1 and 2* (CTT1 and CTT2) [24], [25], and grip strength test [26]. The Box and Block test measures the subjects’ unilateral gross manual dexterity. The Color Trails Test measures executive function and sustained attention. The grip strength test measures hand and forearm strength as a proxy for upper limb strength.

#### 3) Intake Survey:

An intake survey was administered to each subject to determine their baseline affect using the Self-Assessment Manikin (SAM) [27], experience with technology and therapy, feelings about robots and telehealth, and demographic information. For clinical environment subjects, who completed the two-day trial, the SAM was re-administered at the start of the second day to re-assess their affect.

#### 4) Rehabilitation Interactions:

**Fig. 3.**
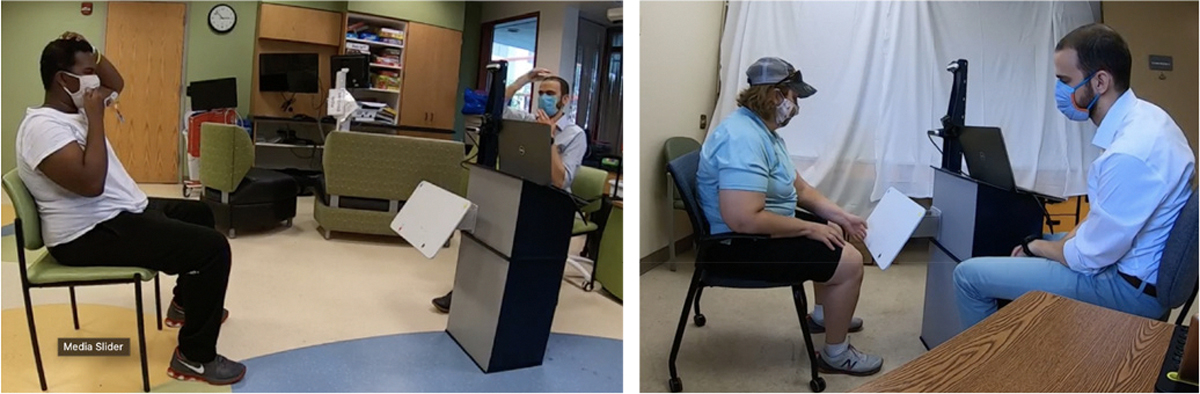
Subjects participating in the face to face (FTF) interaction, Simon says on the left and target touch on the right. Subjects shown provided release to publish images of them.

**Fig. 4.**
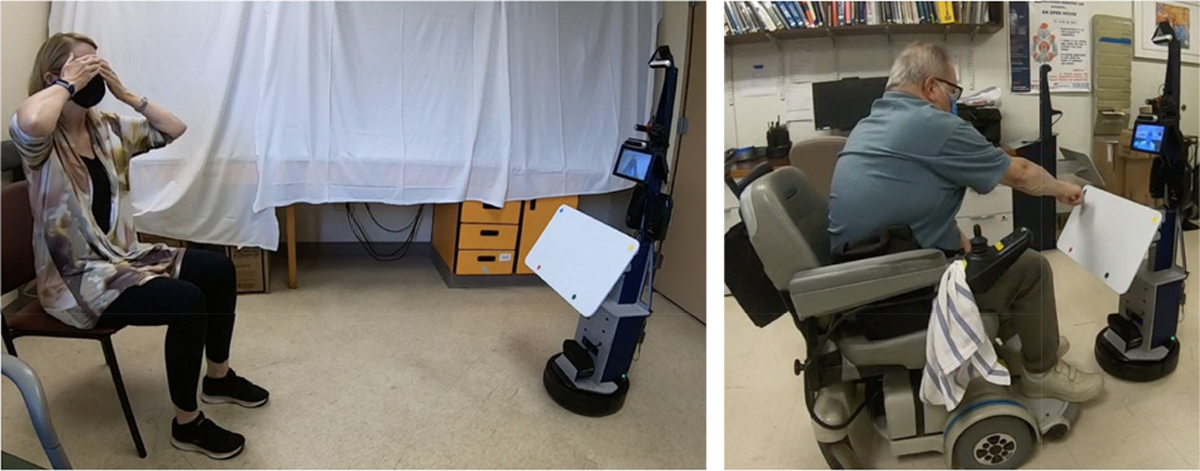
Subjects participating in the classical telepresence (CT) interaction, Simon says on the left and target touch on the right. Subjects shown provided release to publish images of them.

**Fig. 5.**
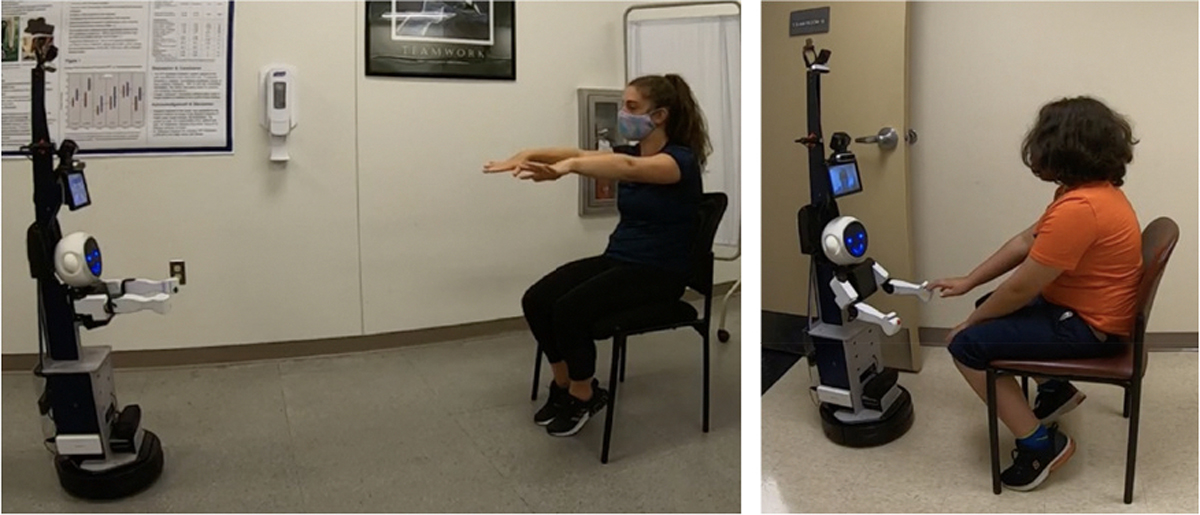
Subjects participating in the social robot augmented telepresence (SRAT) interaction, Simon says on the left and target touch on the right. Subjects shown provided release to publish images of them.

Three rehabilitation interactions were tested: FTF interaction, where the operator is present in the testing environment with the subject, interacting directly with them (fig. 3); CT interaction, where the operator and the subject interact via audio and video (fig. 4); and SRAT interaction, where the subject is introduced to a humanoid robot mounted on the telepresence system by the operator who is virtually present using audio and video. (fig. 5). In the FTF and CT conditions, the operator led interactions, while in the SRAT condition, the robot led the conversation, with the operator interjecting as needed as a secondary facilitator. In the CT and SRAT conditions, the operator was located in a different room from the subject. Throughout the experiments, the subject was recorded by the Intel RealSense cameras mounted on the podium and robot, by a GoPro camera on the podium/robot, and by a GoPro camera located elsewhere in the room which provided a third person perspective.

Previous research indicates that starting with in-person interactions fosters better engagement and understanding of activities during subsequent telepresence sessions with robots. [28]. Therefore, for all subjects, the FTF interaction was completed first. The order of the remaining two interactions (CT and SRAT) was randomly determined using stratified permuted block randomization in blocks of four subjects with strata for the cross of age (4–10, 11–17, 18–49, 50+), motor impairment (impaired, not impaired), and cognitive impairment (impaired, not impaired). At least 10 minutes was allowed to elapse between interactions to allow participants to rest and reduce fatigue, provide a buffer for cognitive recovery (e.g., engagement), and allow for equipment setup for the next interaction modality, ensuring consistency across sessions. The exact duration varied slightly based on setup logistics.

Each interaction condition had two different activities, a Simon says game and then a target touch activity. The Simon says game activity is designed to primarily measures range of motion and reachable workspace, specifically in the context of motions which are relevant to activities of daily living. In the game, the facilitator issued commands and demonstrated tasks (mirrored) for the subject to do. If the command began with “Simon says,” the subject was required to repeat the task; otherwise, they remained in a neutral position. Some tasks were inherently bimanual, like clapping, while others were unimanual and randomly combined to create bimanual tasks, such as reaching with the left arm while touching the left shoulder with the right hand. This combination of tasks enhanced engagement and ensured each game repetition was unique, preventing memorization.

The target touch game assessed motor performance, executive function, and short-term memory. In the game, the facilitator provided a randomized sequence of colors and corresponding hands to touch the dots, with sequences ranging from 1 to 4 touches. In the CT and FTF conditions, four colored dots were positioned near the board’s corners. In the SRAT condition, the humanoid robot’s hands displayed the same colored dots, moving them into positions matching those on the target boards used in the other conditions.

#### 5) Post-Interaction Questionnaire:

After the conclusion of each modality, the subject was presented with a post-interaction survey. To determine the cognitive and physical load placed on the subject during the trial, questions from the NASA Task Load Index (TLX) [29] were used. To determine the subject’s perceived level of pressure during, value attributed to, competence on, and enjoyment of the interaction, scales from the Intrinsic Motivation Inventory (IMI) [30], [31] were used. Questions within the IMI scales were selected based on experience in a pilot trial. Based on results from a pilot trial, the standard IMI scale of seven levels was condensed to five levels to decrease confusion in the target populations.

#### 6) Post-Experiment Survey:

After completing all three interactions, a post-experiment survey were administered to understand modality preference and perceptions on key features of communication, motivation, compliance, and adherence.

### D. Data Analysis

All data analysis was completed using R [32]. Data manipulation was done with dplyr and plots were created using the ggplot package from the tidyverse meta-package [33].

#### 1) Demographics:

Participants are classified into four age groups: young children (4–11 years), teens and young adults (12–20 years), adults (21–64 years), and older adults (≥65 years). Impairments are classified as: no injury, brain injuries (stroke, TBI, CP), peripheral injuries (SCI, amputation, rotator cuff injury, motor development delay), neurodegenerative diseases (Multiple Sclerosis, Parkinson’s), and psychological disorders (autism, conversion disorder).

To determine measured level of impairment across the study sample, the BBT and CTT2 were used. The number of blocks moved in the BBT were age and arm normalized to produce z-scores using healthy population norms from Mathiowetz et al. and Jongbloed-Pereboom et al. [23], [34], [35]. The highest z-score for each arm was taken from three trials per arm and the arm with the maximum score was used for further analysis. Those with weak arm z-scores greater than −1 were determined to have normal gross manual upper extremity dexterity (taken as a proxy for general upper extremity function), those with z-scores of −2 to −1 were taken to have mild impairment, −3 to −2 were taken to have moderate impairment, and less than −3 were taken to have severe impairment.

Similarly, scores from the (Children’s) CTT2 were normalized by age and, for adults, education per the test manual [24], [25] to generate z-scores. For subjects who fell below the published norms for the CTT (z< −3), z-scores were interpolated using the lowest three published z-scores to a minimum z-score of −5. For subjects who were within the administrable age range of the test, but could not complete the CTT, a z-score of −5 was recorded. The levels of impairment cut-off points were simplified from the CTT manual to: greater than −1 as normal, −2 to −1 as mild impairment, −3 to −2 as moderate impairment, and less than −3 as severely impaired.

For reporting, participants with mildly impaired and unimpaired subjects (z≥−2) are grouped together and moderately and severely impaired subjects (z< −2) are grouped together.

Three subjects were too young for the Children’s CTT. One had reported having no impairment and the other two reported having motor impairment only. They were all recorded as having no cognitive impairment. Due to the lack of a valid CTT score, they were excluded from the models that included CTT2 scores (section II-D.3 and II-D.4). One participant felt exhausted during the surveys and withdrew before the final one, thus was excluded from the modality preference analysis models (section III-D). A participant with severe cognitive impairment could not comprehend the surveys, so their results were excluded. They were able to understand the preference question from the final survey, and so their responses to those questions are preserved.

#### 2) Selecting Relevant Factors to Explore:

The central question of this work was whether SRAT would perform better than CT, so the effect of the interaction modality was the primary factor explored. We hypothesized that age, motor function, and cognitive function would affect how subjects experience the various interaction modalities. Additionally, one should expect that the robot operator and the order in which experiments occur could impact results; these are not things we seek to know/quantify, but factors which had to be controlled.

#### 3) Task Load:

The NASA TLX was completed after each interaction. As is typical, we did not perform the optional weighting step as part of the TLX administration. Therefore, we simply averaged the scores for each participant in the TLX to generate an aggregate score for the task load. To understand how these ratings varied between interaction modalities and how relevant factors (age, motor function, cognitive function) affected ratings, a linear mixed model was used. The model was created using the *lme4* package [36] with the equation:

TLX∼interaction.modality*((Age*BBT*CTT2)+experimental.order+robot.operator)+(1∣subject)

Which models the task load (average of TLX ratings) as a linear equation on age, BBT, and CTT, crossed, along with experimental order and robot operator, all crossed with interaction modality. The participant IDs were treated as a random variable to accommodate interactions in the three different modalities. Robot operators were not related as a random effect, since we only have three robot operators. The linear model was checked for the assumption of normality of residuals using QQ-plots of the residuals and the random effects and direct visualization of the residual density plot and for Homoscedasticity with a plot of the residuals against the fitted values.

To understand which terms in the TLX linear model were important, an ANOVA with type III Wald chi-square tests was used (using the *car* package [37]). The significant terms were then visualized to understand how the factors interact to affect the task load. Given the sample size, it was not appropriate to perform any form of post hoc analysis.

#### 4) Competence, Enjoyment, Pressure, and Value:

For the Intrinsic Motivation Inventory (IMI), as suggested by the tool designers [38], we performed a factor analysis to ensure that the individual questions were well aligned with their scales in our sample. One question (“The activities did not hold my attention at all”) had to be dropped from our usage of the IMI because it did not sufficiently load onto its assigned scale (0.27). All other items showed reasonable (*>*0.57) loading on their respective scales. The questions assigned to each scale were averaged to generate scale scores for competence, enjoyment, pressure, and value. To understand how competence, enjoyment, pressure, and value were different among the three interaction modes and how those differences manifested in relation to the important factors of age, cognitive function, and motor function, an analysis method similar to the TLX was used. A single model was constructed independently for each of the four domains of the IMI.

#### 5) Reported Modality Preference:

Subjects ranked their preference for CT or SRAT interaction modalities. This led to six possible orders, which, for analysis, were simplified to the binary of SRAT better than CT or CT better than SRAT, dropping the FTF condition. To determine for which ages, levels of motor function, and levels of cognitive function SRAT is/is not preferred, a generalized linear model with logit linking function was used to convert from probability to log-odds. This model looked at the crossed interactions between age, BBT Scores, and CTT2 Scores and included terms to control for experimental order and robot operator:

srat.better.than.ct∼Age*CTT2*BBT+experimental.order+robot.operator

To determine which factors were important, a Type III ANOVA was used. Results were interpreted using probability plots generated by the logistic function.

## III. Results

### A. Participants

**Fig. 6.**
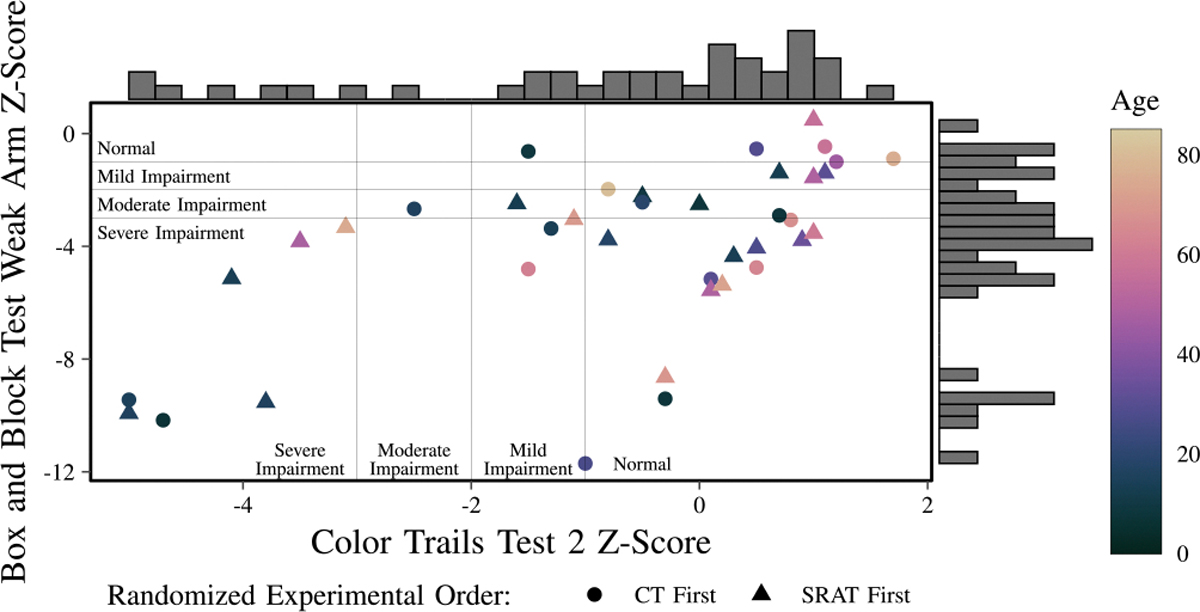
BBT z-scores for subjects’ weak arms and CTT2 z-scores. Age is indicated by color. Levels of impairment for both motor and cognitive impairment are shown. Density for each axis is shown in histograms. Subjects who completed the FTF condition, then CT condition, and finally the SRAT condition, are indicated by a circle. Subjects who did the FTF condition first, followed by the SRAT condition, and finally the CT condition are indicated by a triangle.

The aggregate demographics of the participants can be seen in table I, showing their self-reported impairment, measured impairment (using BBT and CTT2), gender, class of condition, and race and ethnicity. Although some subjects have a condition which could lead to an impairment, they reported not having one, for example, in the case of multiple sclerosis, or having one which has recovered, such as motor impairment from stroke which is no longer present. Subjects had a variety of levels of motor function and cognitive function, as measured by the BBT and CTT2 (fig. 6). The BBT z-scores are used as the sole measure of motor function and CTT2 z-scores are used as the sole measure of cognitive function.

Subjects had positive feelings towards robots with the mean response 4.27 (SD=0.80) of 5. They had low levels of experience with robots (mean=2.14, SD=1.27), mixed prior experience with computers (mean=3.54, SD=1.39), and high experience with smartphones (mean=4.05, SD=1.27) and tablets (mean=3.81, SD=1.33), with higher scores indicating more experience. Thirty-seven (37, 88%) of the subjects reported prior experience making video calls and 19 (45%) reported that they had used video calls for healthcare. Feelings on using video calls for healthcare were mixed, but positive on average (mean=3.68, SD=1.04). Twenty-four (24, 57%) subjects reported receiving therapy: 19 physical therapy, 15 occupational therapy, 8 speech and language pathology, and 6 cognitive and behavioral therapy. They were receiving therapy primarily at hospitals for children (13) and rehabilitation centers (10), along with inpatient facilities (5), outpatient facilities (3), at home (3), at school (2), and at a general hospital (2). Subjects enjoyed their therapy (mean=4.35, SD=0.98) and were highly adherent (mean=4.92, SD=0.28).

On average, subjects started the study with neutral arousal (mean=4.38, SD=2.24, where 1 is excited and 9 is relaxed), neutral dominance (mean=5.54, SD=2.30, where 1 is controlled and 9 is in control), and high valence (mean=2.07, SD=1.47, where 1 is happy and 9 is unhappy).

### B. Task Load

**Fig. 7.**
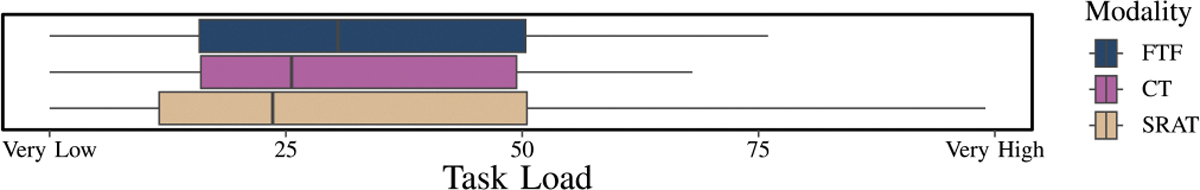
Task load across three interaction modalities is displayed as box and whisker plots, with the box representing the first and third quartiles, the median as a black line, and whiskers extending to values within 1.5 times the interquartile range.

**Fig. 8.**
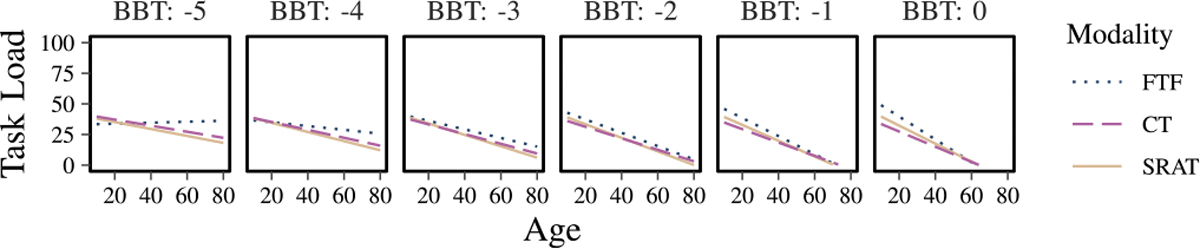
Plots of the estimated results given the model fit to the task load scale, showing the interaction between motor function (BBT z-score) and age in predicting the task load of the three modalities.

Task load was similarly low across all three interaction modalities (fig. 7). The residuals from fitting the task load linear model are approximately normal with no apparent pattern between residuals and fitted values. The ANOVA on the model shows that several factors were significant: age (p=0.007), Age:BBT (p=0.01), interaction modality:BBT (p=0.02), and interaction modality:age:BBT (p=0.05). The highest order of these, which is of interest, is interaction modality:age:BBT, the effects of which are plotted in fig. 8. As can be seen, there are slight differences between age groups and levels of motor function. Specifically, comparing SRAT to CT, the model predicts that people with normal to mildly impaired motor function (BBT*>* −1) and less than 30 years old, will experience slightly higher task load with SRAT than with CT. All other ages/levels of motor function show no visible difference between SRAT and CT.

### C. Competence, Enjoyment, Pressure, and Value

**Fig. 9.**
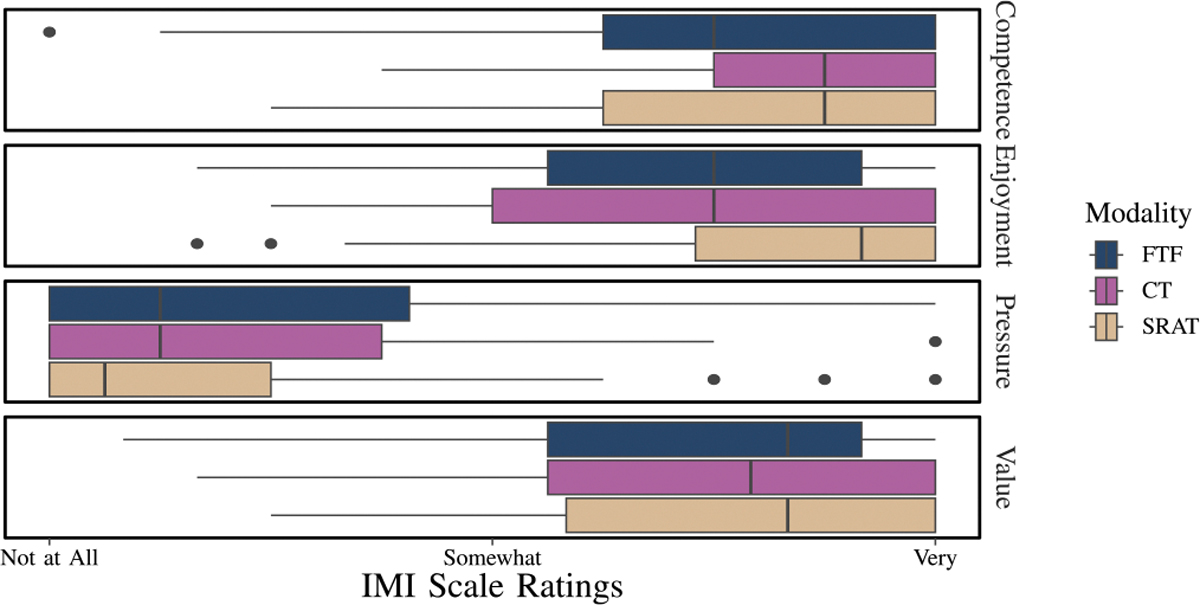
Ratings on the IMI scales for competence, enjoyment, pressure, and value.

The IMI scale ratings are shown in figure 9. Competence scores were high across all three interaction modalities. The ANOVA on the confidence model shows that age:BBT (p=0.008) is a significant contributor to the competence model. Enjoyment scores were also high overall, with the SRAT interaction approximately a quartile higher than the other two interactions. The ANOVA analysis on the model for enjoyment showed no significant factors. Value scores were also high with the ANOVA showing that only age:CTT2 (p=0.048) was a relevant factor. Pressure experienced during each interaction was generally low. The ANOVA for this model did not show any significant factors. The pressure model, due to the limited number of levels in the pressure domains from having a small number of questions, was the least accurate model in that, unlike the other three IMI scales, its residual plot against fitted values showed a non-random distribution. None of the IMI Scale ratings had significant interactions due to interaction modality, suggesting that both SRAT and CT compared favorably to FTF interactions in terms of enjoyment, value, and competence.

**Fig. 10.**
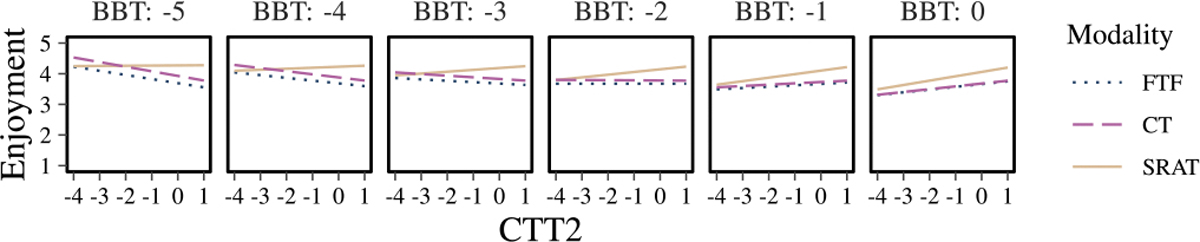
Plots of the estimated results given the model fit to the IMI enjoyment scale, showing the interaction between motor function (BBT z-score) and cognitive function (CTT2 z-score) in predicting the enjoyment level associated with the three modalities.

**Fig. 11.**
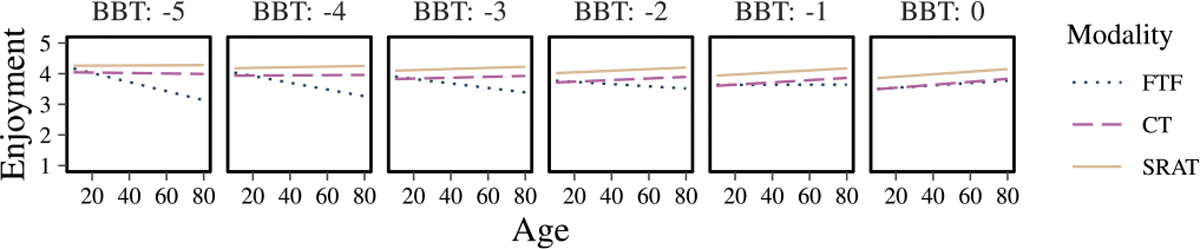
Plots of the estimated results given the model fit to the IMI enjoyment scale, showing the interaction between motor function (BBT z-score) and age in predicting the enjoyment associated with each of the three modalities.

Specifically for the enjoyment model, although the ANOVA did not reveal statistically significant factors, the effect of interaction modality: BBT: CTT2 was observed with a probability of occurring by random chance less than 10% (p=0.081). Figure 10 elucidates the possible interplay between cognition, motor function, and the type of interaction in determining the enjoyment scores. SRAT is more enjoyable than CT among all levels of motor impairment and at normal cognitive function. At and beyond severe cognitive impairment (z< −3), but at all levels of motor function, CT is more enjoyable. Between moderate (z=−2) and severe (z= −3) cognitive impairment, at all levels of motor function, enjoyment is equivalent between conditions. Further investigation of the interaction between motor function, age and interaction modality in understanding enjoyment (fig. 11) revealed that among all ages, SRAT is more enjoyable than CT. These secondary analyses indicate that BBT and CTT2 significantly influence enjoyment, while age is not a determining factor. Notably, enjoyment of SRAT consistently exceeded that of FTF across all ages and levels of BBT and CTT2.

### D. Reported Modality Preference

**Fig. 12.**
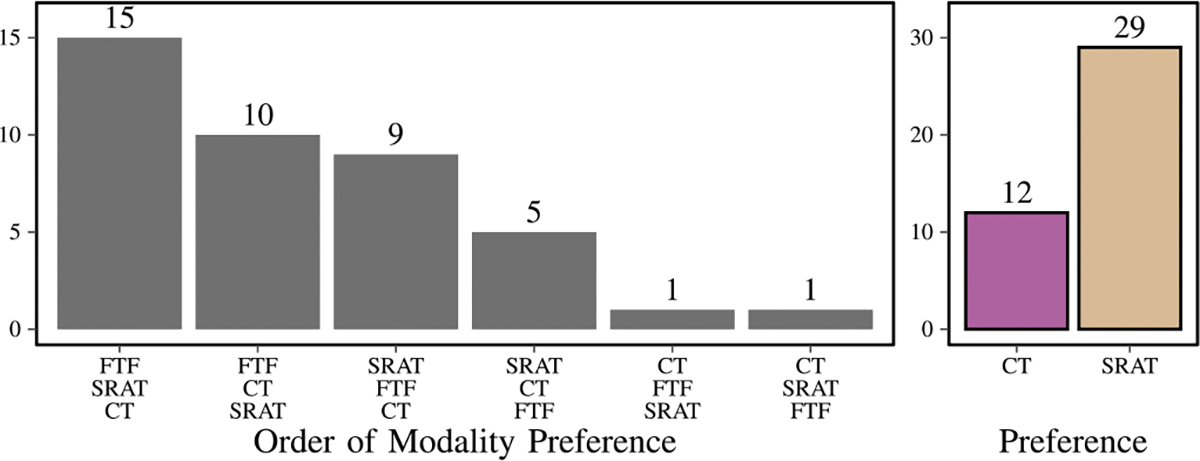
On the left, the ranking of face to face (FTF), classical telepresence (CT), and social robot augmented telepresence (SRAT) by subjects when asked directly to rank them at the conclusion of the study. On the right, the same data, compressed down to only examine the comparison between classical telepresence and social robot augmented telepresence.

Forty-one participants completed the final preference survey. The majority (25 participants, 61%) reported that face-to-face interactions were the best (see fig. 12). SRAT was rated better than CT by 29 participants (71%) and was rated as the best interaction among the three of 14 participants (34%).

**Fig. 13.**
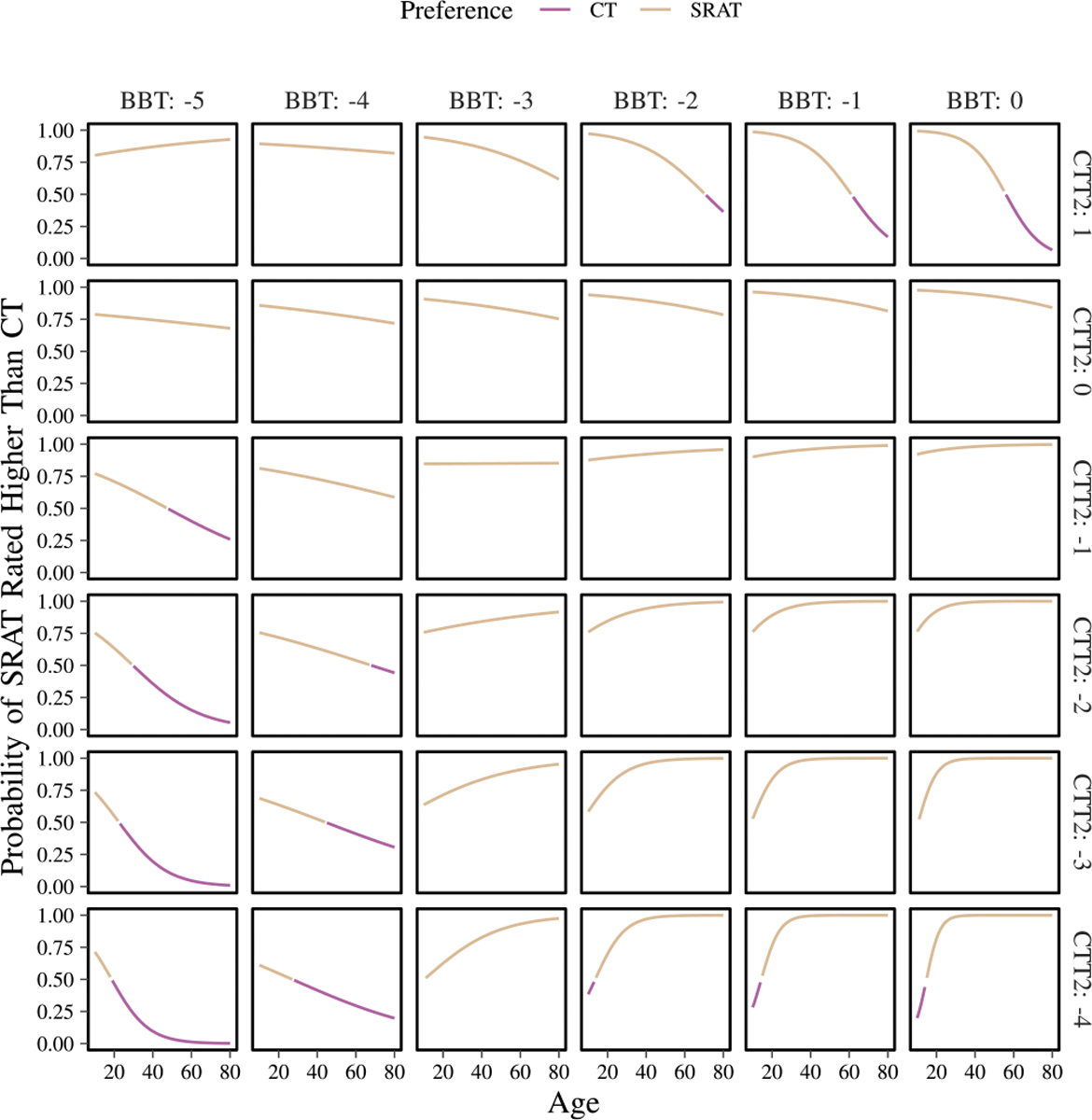
Estimates from the preference model of which people of different ages, levels of cognitive function (CTT2), and levels of motor function (BBT) will prefer SRAT over CT. Probabilities of choosing SRAT over CT by 0.5 or more are shown as preferring SRAT by color, probabilities less than 0.5 are indicated as preferring CT.

The ANOVA on the model for interpreting the preference of subjects, comparing SRAT with CT, had several significant factors: age:CTT2 (p=0.041), CTT2:BBT (p=0.048), age:CTT2:BBT (p=0.048). The interaction of these factors is shown in fig. 13. Among people with high cognitive function (CTT2≥1) and no motor impairment, subjects aged around 60 and older would be expected to choose classical telepresence, as motor impairment increases, this cut-off age increases, exceeding 70 by mild motor impairment (BBT< −2). People with very severe motor impairments (BBT< −4) and moderate cognitive impairment (CTT2< −2) over the age of about 60 would be expected to choose CT over SRAT. As cognitive impairment worsens, the cutoff age shifts younger, reaching less than 30 with very severe cognitive impairment (CTT2< −4). The same trend is found at more severe motor impairment (BBT< −5), where at mild cognitive impairment (CTT2< −1), subjects just over 40 years old would be expected to prefer CT over SRAT and the cutoff once again shifts younger as cognitive impairment increases, reaching approximately 20 years with very severe cognitive impairment (CTT2< −4). In addition, groups of subject with mild or no motor impairment (BBT*>*−2) and severe cognitive impairment (BBT= −4) show an opposite trend, particularly the subjects under 20 years old would choose CT over SRAT. Other groups of subjects are all expected to prefer SRAT, with varying likelihood.

## IV. Discussion

Since the COVID-19 pandemic, the need and call for telemedicine systems has grown [39]. The use of a humanoid robot in addition to a traditional telepresence platform may improve the quality of telerehabilitation based care. This study presented the results comparing the classical method of delivering telerehabilitation (classical telepresence, CT) with a new method using social robot augmented telepresence (SRAT). A tightly controlled study allowed CT and SRAT to be compared across several domains. Subjects who participated covered a broad cross-section of ages, levels of motor function, and levels of cognitive function. By understanding if and how these factors interact with the modalities that we are testing, we hoped to provide a design direction for future development and identify the highest value opportunities for the SRAT modality. Overall, participants had the levels of experience with technology that would be expected from the general population. They reported a low task load when interacting in all three modalities. Only small differences in task load were observed between the SRAT and CT conditions among younger subjects with high motor function (**H1tlx**: not supported). The participants were positive in all interaction modalities, but a majority preferred face-to-face interactions (FTF) more (25 of 41 participants). This is expected and is well reflected in the comment by one of the participants that when FTF interactions are possible, that is preferred, but when they are not, or when they are very costly, that telepresence provides a viable alternative.

The Intrinsic Motivation Inventory (IMI) measures, including competences, pressure, enjoyment, and value, were all positive and did not appear to show any difference between SRAT and CT (**H1c**, **H1p**, **H1e**, and **H1v**: not supported). There was no significant difference between CT and SRAT in terms of enjoyment (**H1e**: not supported). The relationship of perceived enjoyment between the modalities was influenced by cognitive impairment, motor impairment, and age. The highlight is that with no more than mild cognitive impairment, SRAT is either more enjoyable or as enjoyable as CT. With normal motor function, older adults find them equivalent. However, as cognitive impairment increases, some participants find CT to be more enjoyable than SRAT.

Exploration of the mixed models provided some general intuition as to how different people might experience telerehabilitation using a social robot-augmented telepresence compared to the more classical telepresence with a human. The primary takeaway is that the subjects preferred SRAT over CT (29 preferred SRAT, 12 preferred CT) (**H2**: supported). Across all levels of motor and cognitive function, except for those with severe cognitive impairment and motor function from normal to mildly impaired, children (< 20 years of age) are expected to prefer SRAT to CT. Among older adults with severe motor impairment and mild to severe cognitive impairment and adults with high cognitive function and normal motor function, CT is preferred over SRAT. These results mirror the dynamic found for enjoyment. This suggests that SRAT may be particularly beneficial for children and younger adults with moderate impairments, while CT remains a more suitable option for older individuals with severe cognitive or motor deficits. Clearly there is an impact on preference based on impairment level and age and, as expected, older adults are less likely to prefer SRAT, a new unfamiliar technology.

One possible reason for the dynamic observed in both modality preference and enjoyment across modalities is that interactions by robot are less resilient to perturbation as a result of cognitive impairment, causing CT, with a flexible human as the center of the interaction, to be more enjoyable. This is not necessarily a reflection of the concept of SRAT, but instead on the specific form of SRAT presented here, in which interactions were very robot centric. It is also possible that with a certain level of cognitive impairment, a robot is simply too foreign for a person to understand. However, our study suggest that people with severe cognitive impairments older than 50 years are expected to enjoy SRAT more than CT. This finding highlights the need for further investigation into how SRAT can be adapted to better support older adults with cognitive deficits, potentially through enhancements in verbal and gesture-based interaction and simplified task structures. Supporting this, Wu et al. [40] showed that seniors with mild impairment can learn how to use the Kompaï robot when given streamlined instructions and multimodal prompts, albeit over longer training periods.

Secondary analyses of the enjoyment model also suggested a higher perceived enjoyment towards SRAT compared to the FTF modality, regardless of the participants’ age. Lukasik et al. showed that the psychosocial interventions provided by SAR were accepted similarly across different age groups [41]. In general, older people are expected to reject robots, as suggested by the results of Chien et al., in which older adults had a more implicit negative attitude towards robots before interacting with them [42]. The also revealed that both young and old adults had more explicit positive attitudes towards robots after interacting with them [42].

Although our data show some groups of older people preferring CT over SRAT, it does not show a strong rejection of SRAT by elders and among some groups of elders, SRAT was more enjoyable than CT. Notably, the degree of motor and cognitive impairment seems to be a stronger determinant of preference than age alone, suggesting that individual functional capacity plays a key role in how well participants engage with SRAT. In our finding, we observed greater enjoyment of SRAT compared to the interaction modality of FTF when considering age interaction with motor impairment of the upper limb (fig. 11). This result could appear as initial excitement and curiosity that arise when participants encounter this new robotic system, a phenomenon known as the novelty effect [43]. Our finding that SRAT is preferable and more enjoyable than CT might also be due to the stronger novelty effect generated by the physical presence of the embodied robot compared to that of non-embodied robotic system such as the CT modality. This phenomenon suggests that the effect tends to fade over time as individuals become accustomed to the robot. When SRAT was deployed in the community with older adults, older adults maintained their interest in this system [44]. These results were also supported by a study by Feingold Polak and Levy-Tzedek showing a trend of acceptance of SAR by stroke patients following a long-term interaction [45]. Feingold Polak and Levy-Tzedek also suggested possible guidelines that could help maintain long-term engagement and effectiveness, such as personalization of the SAR system and adaptability of the interaction. To determine whether the novelty effect occurs with the SRAT interaction, future research is required on the longitudinal effects of this robotic system in rehabilitation settings. Additionally, future iterations should focus on customizing SRAT for different age groups and impairment levels to enhance accessibility and engagement.

The Flo SRAT platform had some challenges. Several subjects said that the humanoid robot is distracting. To some extent, the robot is meant to grab the patient’s attention. But when it overshadows the activities to be done, that is not a good thing. As robots become more ubiquitous in society, the problem of being distracting will decrease. Even over the course of the trial, some subjects suggest that the distraction decreased, that the robot was most distracting at the beginning of the trial. Subjects also complained about the fidelity of the arm movements on Flo, the quality of the voice, and the system’s responsiveness. All very reasonable observations given the nature of the demonstration system. What is exciting is that, even with all the challenges inherent in an early prototype, SRAT was still preferred over CT. Clearly SRAT is worth further exploration as a way to improve telerehabilitation to bridge the growing gap in care. We anticipate that advances in automation and adaptive robot operation will enhance SRAT by reducing operator workload and improving patient responsiveness. Future iterations could integrate gesture recognition, natural language processing, real-time engagement monitoring, and personalized feedback based on cognitive and motor abilities. These improvements will make SRAT more autonomous, enhancing patient engagement and rehabilitation outcomes [46], [47], [48].

This is one of the first studies to look at the complex intersection of age, cognition and motor function on social robot preference. Our probability models suggest some significant interactions around preferences and should serve to provoke thought on how different people interact with robots and via telepresence and should be taken as an invitation for further exploration. Despite these promising results, there were several limitations to our study. The sample size for this study is good for both the fields of social robotics and rehabilitation robotics. The sample is broad and honest. However, the sample size is still small to draw definitive conclusions on how different groups react to SRAT. The ratio of samples to parameters in the generalized linear models used to understand how different groups react to SRAT versus CT is non-favorable, which limits the interpretation which can be made from those studies. Linear models were used throughout, this is standard in the field (the ANOVA is a special case of the generalized linear model), however, other more flexible models may be more appropriate. More flexible models would however require a larger sample to fit appropriately. Another challenge is the presence of holes in age/level of motor and cognitive function, there was only one subject with moderate cognitive impairment and no subjects with normal or mild motor impairment and cognitive impairment (fig. 6). The models likely do not adequately capture those subpopulations, indicating a need for larger studies. Additionally, the absence of key demographic subgroups (e.g., individuals with mild motor impairments and moderate to severe cognitive impairments) limits the generalizability of our findings. Of course, other factors, like the affect of subjects, prior telepresence experience, feelings towards robots, etc. could also (probably do) impact results. Participants who volunteered for the study may have had a higher baseline interest in technology or robotics, potentially introducing selection bias and skewing preferences toward SRAT. These factors suggest that the observed trends should be interpreted with caution. However, these are not the factors that we can design around as roboticists. We cannot make robots and specify that they are only supposed to be used with happy subjects who are alert. We can design robots that should interact with people in the populations that need them as they are. Our results suggest that age, motor function and cognitive function of the population using the robots can influence their telerehabilitation preference and their subsequent enjoyment of the rehab session. Thus, it is very reasonable to design a robot differently for adults versus children or people with high cognitive function vs low cognitive function. Nevertheless, future research should focus on expanding sample diversity and size to validate these findings across broader populations, particularly in older adults with varying levels of cognitive and motor impairments.

Other limitations of our experimental setup include the lack of physiological monitoring (e.g., heart rate, perceived soreness) and the absence of a standardized rest interval between modalities. Although we enforced a minimum ten-minute break, actual recovery periods varied, potentially leaving some participants with residual physical or mental fatigue that could shorten attention spans and reduce robot acceptability [49]. Nonetheless, mean task-load ratings were comparable across modalities (fig. 7), albeit with greater variance in the SRAT condition. Another source of variability was the study schedule: children aged 4–18 in the clinical environment completed FTF on day 1 and both CT and SRAT on day 2, whereas other participants completed all sessions in a single day. While such timescale discrepancies might influence user responses, they are unlikely to account for our findings, since those in the two-day protocol were generally more severely impaired. We therefore believe that age, motor function, and cognitive status had a stronger impact on outcomes than these scheduling differences (fig. 7, fig. 8, and fig. 11). Additionally, performance metrics—such as accuracy and response time in tasks like Simon Says—were not formally recorded; instead, experimenters adjusted robot behavior in real time to preserve a naturalistic, adaptive experience. Future work should integrate objective performance tracking to compare accuracy and success rates across modalities. Likewise, we did not conduct formal hearing or vision assessments; prescreening simply confirmed that participants could “follow instructions,” and post-interaction questionnaires (“How well did you understand your tasks?”) indicated that most subjects felt they understood the activities. Future studies could employ standardized sensory screenings to clarify the impact of any impairments on interaction quality.

## V. Conclusion

This work introduces and evaluates a Social Robot-Augmented Telepresence (SRAT) system as an innovative platform for remote rehabilitation. In a controlled study spanning diverse age groups and levels of impairment, SRAT was preferred over classical telepresence (CT) by most participants, particularly those with intact or mildly impaired cognitive function. Interaction quality, task load, and user motivation were comparable across modalities, indicating the feasibility and acceptability of SRAT in telerehabilitation contexts. Importantly, preference for SRAT was modulated by age, motor ability, and cognition, underscoring the need for user-centered design in future iterations. Despite limitations in sample size and prototype fidelity, these findings support SRAT as a promising tool for expanding access to care. Future research should focus on adaptive automation and longitudinal evaluation to optimize its clinical utility.

## Supplementary Material

supp1-3592020

## Figures and Tables

**TABLE I:** Subject Demographics. Percentages Are Shown Per Column for Each Category. Measured Impairment Is Shown for Subjects Measured to Have Moderate or Severe Impairment. Percentage Not Show for Race/Ethnicity Due to Multiple Selection

		Young Children N = 9	Teens-Young Adults N = 11	Adults N= 15	Older Adults N = 7	Sum N = 42

**Reported Impairment**	Cognitive	0 (0%)	1 (9.1%)	0 (0%)	1 (14%)	2 (4.8%)
	Motor	4 (44%)	5 (45%)	4 (27%)	4 (57%)	17 (40%)
	Motor and Cognitive	3 (33%)	3 (27%)	5 (33%)	2 (29%)	13 (31%)
	None	2 (22%)	2 (18%)	6 (40%)	0 (0%)	10 (24%)

**Measured Impairment**	Motor	6 (67%)	5 (45%)	8 (53%)	4 (57%)	23 (55%)
	Motor and Cognitive	1 (11%)	5 (45%)	1 (6.7%)	1 (14%)	8 (19%)
	None	2 (22%)	1 (9.1%)	6 (40%)	2 (29%)	11 (26%)

**Gender**	Male	6 (67%)	7 (64%)	5 (33%)	1 (14%)	19 (45%)
	Female	3 (33%)	4 (36%)	10 (67%)	6 (86%)	23 (55%)

**Class of Condition**	Brain Injury	2 (22%)	5 (50%)	6 (40%)	4 (57%)	17 (41%)
	Neurodegenerative Disorder	0 (0%)	0 (0%)	5 (33%)	2 (29%)	7 (17)%
	Peripheral Injury	2 (22%)	2 (20%)	2 (13%)	1 (14%)	7 (17%)
	Psychological Disorder	1 (11%)	1 (10%)	0 (0%)	0 (0%)	2 (4.9%)
	No Injury	2 (22%)	1 (10%)	2 (13%)	0 (0%)	5 (12%)
	Unknown	1 (11%)	1 (10%)	0 (0%)	0 (0%)	2 (4.9%)
	Other	1 (11%)	0 (0%)	0 (0%)	0 (0%)	1 (2.4%)

**Race/Ethnicity**	American Indian or Alaska Native	1	0	1	0	2
	Asian	0	0	2	0	2
	Black or African American	3	5	3	1	12
	Hispanic or Latino	1	0	1	0	2
	Middle Eastern or North African	0	1	0	0	1
	White	5	4	13	6	28
	Prefer not to answer	0	2	0	0	2
