# Supplementary material for: Age, Motor Function, and Cognitive Function Influence Preferences for Telerehabilitation Mediated by a Social Robot Augmented With Telepresence
Source: IEEE Trans Neural Syst Rehabil Eng. Author manuscript; Available in PMC 2025 Oct 17. (PMC12532247; doi:10.1109/TNSRE.2025.3592020)
Supplement: supp1-3592020 [file NIHMS2101619-supplement-supp1-3592020.pdf]

Age, Motor Function, and Cognitive Function Influence Preferences for Telerehabilitation Mediated by a Social Robot Augmented with Telepresence - Supplementary Materials

Michael J. Sobrepera†, Anh T. Nguyen, *Member, IEEE*†, Ajay Anand, Laura A. Prosser, Sally H. Evans, Michelle J. Johnson, *Member, IEEE*

This supplementary document complements the main paper, providing tables that summarize surveys, questionnaires, and game prompts. Tables I, II, III, and IV detail the surveys used at various experimental stages, while Table V lists the prompts for the Simon Says game. We also provide the qualitative feedback from the participants in section I.

TABLE I
PRE-SCREEN FORM, EXCLUDING CONTACT INFORMATION QUESTIONS

Question	Input Format
Date of Birth	Date
Gender	Gender
Have you had/do you have any of:	Multiple Choice: None, Other, Stroke, Heart Attack, Cerebral Palsy, Traumatic Brain Injury, Multiple Sclerosis, Parkinsons, Spinal Cord Injury, Peripheral Nerve Injury
Which sides of your body did your stroke (or other brain trauma) affect (often the opposite of the side of the brain the stroke occurred on)	Multiple Choice: Left, Right
Does the subject have a motor impairment?	Yes/No
What is their motor impairment?	Text Entry
Does the subject have a cognitive impairment?	Yes/No
What is their cognitive impairment?	Text Entry
Other notes on diagnostics	Text Entry
Arm Function	No, Somewhat, Yes, No answer
Can touch head?	
Can reach arms out in front?	
Can reach arms out to the side?	
Sitting Function	Yes/No
Does the subject use a wheelchair?	
Can the subject sit without help, with free movement of arms (trunk support ok)?	
Can the subject follow instructions?	No, Somewhat, Yes, No answer

I. Responses to Open Ended Questions

During each post-interaction survey and in the final survey, the subjects were asked for other thoughts on their experience. Some of the responses provide further context on the data and interesting points to ponder:

One of the subjects summed up the utility of telehealth: “If I’m remote this is a good way to go about doing rehabilitation but if I can access a physical person, I would prefer that, but if this were a lower cost alternative, I might still choose tele-rehab” (49 years old, motor impaired, brain injury).

Subjects reported things they liked about Flo. A subject said that Flo was “Very cute and adorable” (16 years old, motor and cog impaired, brain injury). Another thought the system was “Gorgeous” (70 years old, motor impaired, neurodegenerative disorder). One subject named the robot John. They were very excited to interact with technology in general. After the CT condition, they reported “I enjoyed that but I really love John” (10 years old, motor impaired, psychological disorder). Another subject reached out to the study team after the trial to share some further thoughts on the utility of a social robot: “It occurred to me that Flo is perfect for working with folks with disabilities because she is entirely non-judgmental. She is unaware of age, race, intelligence, etc” (81 years old, unimpaired, brain injury).

Some subjects articulated why they preferred CT over SRAT. One said “It’s [(CT)] better than with the robot, easier, more natural.” (28 years old, motor impaired, peripheral injury). Some subjects found the humanoid distracting, one said “I was a little distracted by the robot since I’m so used to one on one therapy” (29 years old, no impairment) and another reported “In the beginning I was kinda distracted by the robot’s movement and how different it was from a human’s, it was distracting at the beginning” (30 years old, motor impaired, brain injury). Yet another said: “For myself I was so distracted by watching the robot move that I was unable to concentrate on the task that I was given. The robot was very fascinating, but it distracted from doing the actual rehabilitation activities”, but later said “this could be very useful for telehealth. [..] Robots like this could really change the way telehealth and telerehabilitation is done in the future.” (49 years old, unimpaired, neurodegenerative disorder). One subject was intimidated by the robot “It’s a little bit scary to me because I’m not used to it [(robots)], I’m more used to people but I think I can get used to it” but felt that others would not be intimidated: “All in all a good experience and a whole lot of other people in my group would not be intimidated by the robot” (62 years old, motor impaired, brain injury). Another subject felt that the robot made more sense for pediatric use “The robot would probably be more helpful for younger people (or children)” and went on to say that “I’m very extroverted and would much rather have a human being to interact with” (56 years old, unimpaired, neurodegenerative disorder).

A subject complained that the social robot was not as responsive as the operator: “The robot didn’t give feedback as often as [the operator] did in the classical telepresence. Had to wait for her to say go which challenged memory” (64 years old, motor impaired, brain injury). Similarly, another subject felt that the robot did not demonstrate activities long enough: “During Simon says, in classical telepresence or in person, [the operator] kept doing the action such as swinging his arm but with Flo, she stopped so it might cause confusions on whether to continue doing the action” (29 years old, unimpaired).

A few subjects reported challenges with the voice used on Flo. One said “It was a little harder, being able to hear it from a human was a little easier. Hearing and knowing it was a robot made it a little harder.” But then reported that working via CT led to more errors: “I messed up more this time” (14 years old, motor impaired, no injury). Similarly, another subject said: “It would be a better if the voice wasn’t choppy, it sounds like a computer. It would be better if it sounded like Siri and Alexa, more like a human” (19 years old, motor impaired, psychological disorder). However, other subjects liked the voice for the robot: “I liked the robot, I liked the faces, voices have matured and grown up vs the older (synthesized) voices which were very monotone” (49 years old, motor impaired, brain injury). Another subject thought the robot voice was so good that it was higher quality than a human over telepresence: “Could hear it better, very understandable. Maybe Flo’s voice is clearer than [the operator’s]” (16 years old, motor and cog impaired, brain injury).

Subjects also observed that Flo’s range of motion is not quite sufficient to complete all of the tasks and sometimes its arms are a bit too large: “while doing some of the physical stuff it should actually be able to get there (like reaching shoulder, instead of that it collided hands)” (19 years old, motor impaired, psychological disorder), “Major changes that would help her would be to have the arms not hit/clunk/smaller hands when covering mouth” (34 years old, motor impaired, brain injury).

One subject felt the robot should be height adjustable to be able to sit at eye level with everyone who interacts with it. Similarly, they requested that the target touch board be able to move up and down (19 years old, motor impaired, psychological disorder).

A number of subjects commented on the small screen size on the Flo system: “The screen was kinda small, would have been nice to have a larger screen where I could see all of his [(the operator’s)] body” (30 years old, motor impaired, brain injury), “It was hard to tell the difference between shoulder flexion and abduction in the small screen during the telepresence” (29 years old, no impairment), “Classical telepresence would be better with at least the full torso in view” (31 years old, no impairment), “If the screen on the classical telepresence robot was a little bigger it would increase its efficiency” (64 years old, motor impaired, brain injury), “Screen was too small to see the therapist’s arms sometimes” (73 years old, motor impaired, brain injury).

Subjects also shared ways in in which the activities provided a challenge for them. Some subjects found it challenging to keep straight which hand was their left and right hand for the target touch activity. This was noted for a subject who was four

years old and one who was eight years old, both with motor impairments. More generally, multiple subjects reported on the cognitive load presented by the activities (after the FTF condition): “Effort was mostly having to think. Nothing hard, just had to listen before moving. Put your mind to it.” (16 years old, motor and cog impaired, brain injury), “[the activities] make you think about what you are doing, you have to use your brain a lot” (19 years old, motor impaired, psychological disorder).

Two subjects recommended alternative ways in which to use Flo. A subject saw potential to use the social robot in triadic interactions in person: “It would be nice to have the robot with the human as an aid [in-person]” (30 years old, motor impairment, brain injury). A different subject was interested in using the humanoid robot by itself: “Would be interesting to not be able to see [operator’s] face when working with Flo” (64 years old, motor impaired, brain injury).

TABLE II
INTAKE SURVEY

Question	Input Format
How are you feeling right now? (Self-Assessment Manikin)	Images from SAM with 9 steps:
Affect	1: Happy – 9: Unhappy
Arousal	1: Excited – 9: Relaxed/Sleepy
Dominance	1: Dominant/In Control – 9: Submissive/Being Controlled
How do you feel about robots?	Likert: 1: Very Negative – 5: Very Positive
Please rate your level of experience with the following:	Likert: 1: No Experience – 5: Very High Experience
Computers	
Tablets	
Smartphones	
Robots	
Do you currently receive therapy?	Yes/No
Where do you currently receive therapy? (If currently receiving therapy)	Checkboxes
School	
Hospital for children	
General hospital	
Elder care hospital	
Rehab center	
Elder care home	
Community center	
At home	
Inpatient facility	
Outpatient facility	
Other	
What other locations? (If Other selected)	Text Entry
What kind of therapy do you receive? (If currently receiving therapy)	True/False Checkbox
Physical Therapy	
Occupational Therapy	
Speech and Language Pathology	
Cognitive Behavioral	
Other	
What other types? (If Other selected)	Text Entry
How much do you enjoy your current therapy? (If currently receiving therapy)	Likert: 1: Not at all – 5: Very much
How often do you do the therapy you are supposed to do? (If currently receiving therapy)	Likert: 1: Never – 5: Always
Do you take any mood or focus-altering medications?	Yes/No
Which mood or focus-altering medications do you take? (If taking)	Text Entry
Have you ever done a video call?	Yes/No
Have you ever done a video call for healthcare?	Yes/No
How do you feel about using video calls for healthcare?	Likert: 1: Very negative – 5: Very positive
How would you describe yourself? (Select all that apply)	Checkboxes
American Indian or Alaska Native	
Asian	
Hispanic or Latino	
Black or African American	
Middle Eastern or North African	
White	
Native Hawaiian or other Pacific Islander	
other	
prefer not to answer	
Please specify other (if other selected):	Text Entry

TABLE III
POST INTERACTION SURVEY

Question	Input Format
Please answer the following questions based on the interaction you just had using the sliders:	Slider:
How well did you understand what you were supposed to do?	Not at all – Perfectly
Would you want to have this interaction again?	Not at all – Very much
How safe did you feel during the interaction?	Not at all safe – Very safe
Mental Demand: How mentally demanding was the interaction?	Very Low – Very High
Physical Demand: How physically demanding was the interaction?	Very Low – Very High
Performance: How well did you perform the tasks you were asked to do?	Failure – Perfect
Effort: How hard did you have to work to perform the activities asked of you?	Very low – Very high
Frustration: How insecure, discouraged, irritated, stressed, and annoyed were you?	Not at all – Very much
Enjoyment: How much did you enjoy the interaction?	Not at all – Very much
For each of the following statements, please indicate how true it is for you, based on the interaction you just had and activities you just completed, using the following scale:	Likert: 1: not at all true – 3: somewhat true – 5: very true
I was anxious while doing the activities	
The activities were fun to do	
I believe the activities could be of some value to me	
I would describe the activities as very interesting	
I was very relaxed in doing the activities	
I think that doing these activities is useful for rehab	
I thought the activities were quite enjoyable	
I think doing these activities could help me to improve my arm function	
This was an activity that I couldn't do very well	
The activities did not hold my attention at all	
I am satisfied with my performance at these tasks	
This was an effective method of doing rehab	
Do you have any other comments or thoughts about this interaction?	Text entry

TABLE IV
FINAL SURVEY

Question	Input Format
Please rank which interaction you thought was best, second best, and worst:	Best, Second best, Third best:
Face-to-face	
Telepresence + Social Robot	
Classical Telepresence	
Do you think telehealth would change how you manage your health and medical needs if you and your clinician used it?	Likert: 1 Not at all – 5: Very much
Would you follow your doctor's/therapist's/nurse's advice less or more if they worked with a telehealth system?	Likert: 1: Much Less – 3: No Change – 5: Much More
Would video visits be a convenient form of healthcare delivery for you?	Likert: 1: No – 5: Yes
Please rate how you believe that using the humanoid robot (like Lil'Flo, with arms and a head) with video telepresence will compare to using video telepresence alone:	Likert: 1: Much better with humanoid – 3: No difference – 5: Much better without humanoid
Communication between me and the clinician	
My motivation to do rehab activities	
My compliance with instructions during interactions	
My adherence to treatment plans after interactions	
What locations do you think Lil'Flo could be deployed in?	Checkboxes
Rural outpatient clinics	
Rural inpatient clinics	
Elder care facilities	
Schools	
Patient homes	
Community centers	
Urban inpatient clinics	
Urban outpatient clinics	
None	
Other	
What other locations? (If other selected)	Text input
Are there other activities which you would like to do with Lil'Flo?	Text input
Please rate your impression of Lil'Flo on these scales:	5 Element Likert Scales from 1 – 5
Dislike – Like	
Unfriendly – Friendly	
Unkind – Kind	
Unpleasant – Pleasant	
Awful – Nice	
Do you have any other comments or feedback?	Text entry
Before this study, did you have any prior experience with Lil'Flo?	Checkboxes
No prior knowledge	
I have read a paper on the system	
I have seen the system in person	
I have used the system	
I have some other experience with system	
What other prior experience? (If other selected)	Text entry

TABLE V
MOVEMENTS USED IN THE SIMON SAYS GAME

Movements
Clap your hands
Raise your arms up over your head
Touch your right hand to your left shoulder
Touch your left hand to your right shoulder
Reach forward with your arms
Cover your eyes with your hands
Touch your mouth with your right hand
Touch your mouth with your left hand
Touch your head with your right hand
Touch your head with your left hand
Reach to the side with your right arm
Reach to the side with your left arm
Wave with your right arm
Wave with your left arm
Rotate your right arm like me
Rotate your left arm like me
Swing your right arm up and down like this
Swing your left arm up and down like this
Swing your right arm to the side like this
Swing your left arm to the side like this
